# Clinical characteristics and associated risk factors for diminished ovarian reserve among Chinese women: a matched case-control study

**DOI:** 10.3389/fendo.2026.1767835

**Published:** 2026-02-26

**Authors:** Fan Zhao, Penghao Li, Ruobing Mei, Chongbi Huang, Dongsen Hu, Tony Cheung, Yajiao Lu, Pulin Luo, Lucas Gonzalo Garay, Ying Yang, Dandan Zhao, Juan Yang, Jing Li, Leesa Lin

**Affiliations:** 1 Department of Epidemiology and Statistics, West China School of Public Health and West China Fourth Hospital, Sichuan University, Chengdu, China; 2 Sichuan Jinxin Xinan Women’s and Children’s Hospital, Chengdu, China; 3 Department of Infectious Disease Epidemiology, London School of Hygiene and Tropical Medicine, London, United Kingdom; 4 Laboratory of Data Discovery for Health Limited (D24H), Hong Kong, Hong Kong SAR, China; 5 Moonrise Initiative, Hong Kong, Hong Kong SAR, China; 6 WHO Collaborating Centre for Infectious Disease Epidemiology and Control, School of Public Health, LKS Faculty of Medicine, The University of Hong Kong, Hong Kong, Hong Kong SAR, China

**Keywords:** age, China, diminished ovarian reserve, matched case–control study, risk factors

## Abstract

**Background:**

Diminished ovarian reserve (DOR) has emerged as a significant reproductive challenge and a broader societal concern. Most previous studies have focused on ovarian reserve markers, while limited research has examined DOR as a primary outcome, and the potential association between TORCH infections (toxoplasmosis, others, rubella, cytomegalovirus, herpes) and DOR risk remains unclear.

**Methods:**

A matched case–control study was conducted among women aged 20–47 years who sought assisted reproductive technology at a maternity hospital in Sichuan, China, between January 2022 and August 2024. DOR was diagnosed according to the Consensus on clinical diagnosis and management of diminished ovarian reserve from China. Age-matched controls (1:1) with normal ovarian reserve were selected. Conditional logistic regression was used to identify factors associated with DOR, with multivariable models adjusting for confounders. Subgroup analyses by age and body mass index (BMI) were conducted to examine robustness and effect modification.

**Results:**

A total of 3,751 DOR cases were matched to 3,751 controls (median age: 36 years). DOR group had significantly higher FSH, E2, and LH levels (P < 0.01), and lower AFC, AMH, PRL, and T levels (P < 0.001) compared to controls. Multivariable logistic regression showed that non-Han ethnicity (OR = 1.278, 95% CI: 1.115–1.466), manual labor (OR = 1.181, 95% CI: 1.002–1.392), obesity (OR = 1.316, 95% CI: 1.044–1.660), light menstrual flow (OR = 1.262, 95% CI: 1.111–1.435), and T. gondii infection (OR = 2.292, 95% CI: 1.683–3.122) were independently associated with DOR. In women aged 20–35 years, ≥2 pregnancies (OR = 0.712, 95% CI: 0.615–0.824), and infections with T. gondii (OR = 23.750, 95% CI: 13.330-42.316), CMV (OR = 8.189, 95% CI: 5.821-11.521), and RV (OR = 8.132, 95% CI: 5.806-11.390) were strongly associated with DOR, with no such associations observed in the 36–47 years group. Significant age interactions were detected (P < 0.05).

**Conclusion:**

Ethnicity, obesity, menstrual flow, pregnancy history, and TORCH infections were significantly associated with DOR, with age-related effect modification observed for pregnancy history and infections. Prospective studies are needed to elucidate the underlying mechanisms, particularly the role of infections and immune response.

## Introduction

Approximately 10–20% of couples in the world are infertile (1). In China, the estimated infertility prevalence reaches 15.5% in the reproductive-aged female population (2). The most common causes of infertility are ovulatory dysfunction, male factor infertility, and tubal disease (3). Diminished ovarian reserve (DOR), a reflection of reproductive potential, is a significant cause of female infertility, accounting for about 10% of infertility cases (4). Ovarian reserve refers to the quantity and quality of the remaining oocytes in the ovaries. DOR describes women of reproductive age having menses whose response to ovarian stimulation or fecundity is reduced compared with women of comparable age (3, 5).

In recent years, DOR prevalence has increased and now tends to occur at younger ages, with the reported prevalence of DOR varying between 10 and 35%, depending on differences in the definition of DOR (7). DOR not only leads to reproductive dysfunctions such as menstrual irregularities, recurrent miscarriage, and infertility, but may also result in premature menopause, thereby adversely affecting women’s quality of life (8, 9).

Although age has been established as a well-known independent risk factor, the etiology of DOR remains elusive (6, 10). As a complex clinical condition, ovarian reserve can be influenced by a range of factors, including medical causes, genetic variables, environmental exposures, and unidentified contributors (6, 11). With an increasing number of women delaying marriage and childbirth, DOR has emerged as an intractable and complex issue for women wishing to conceive, as well as a broader societal challenge for future generations (12). While previous studies have primarily focused on ovarian reserve markers such as anti-Müllerian hormone (AMH) and antral follicle count (AFC) (13–15), limited research has used DOR as a primary outcome, and the potential association between TORCH infections and DOR risk remains unclear.

To address this knowledge gap, we conducted a large-scale, age-matched case–control study to comprehensively characterize the clinical features of DOR and identify potential risk factors, including TORCH infections (toxoplasmosis, others, rubella, cytomegalovirus, herpes) and DOR risk remains unclear.

## Materials and methods

### Ethical approval

This study was approved by the Ethics Committee of West China Fourth Hospital and West China School of Public Health (Approval No. Gwll2024143). The data were accessed for research purposes from June 1, 2024 and August 7, 2024. The authors did not have access to information that could identify individual participants at any stage of the study. The requirement for informed consent was waived by the ethics committee as the study involved the analysis of existing, anonymized patient data.

### Study design and participants

**Figure 1:**
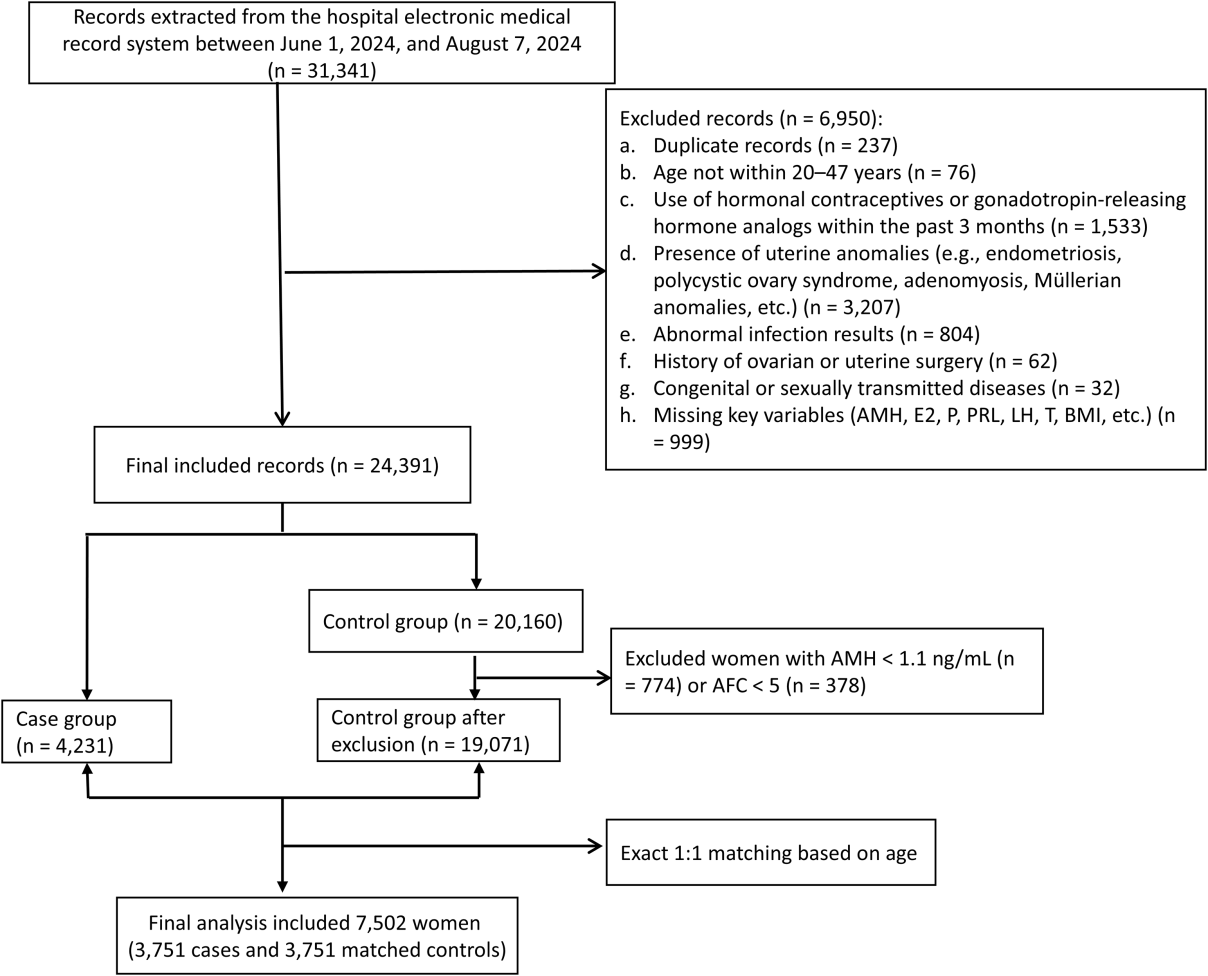
Flow chart of participant selection.

Research participants were women aged 20–47 years who sought assisted reproductive technology (ART) treatment at maternity hospital in Sichuan, China, between January 2022 and August 2024. According to the Consensus on Clinical Diagnosis and Management of Diminished Ovarian Reserve (16), the diagnosis of DOR was based on a comprehensive evaluation of ovarian reserve function by gynecologists, integrating AMH levels, AFC, and follicle-stimulating hormone (FSH) levels. DOR was diagnosed when at least two of the following criteria were fulfilled: (a) AMH < 1.1 ng/mL; (b) bilateral AFC < 5; and (c) FSH ≥ 10 IU/L on two consecutive menstrual cycles. Women aged 20–47 years diagnosed with DOR were included in the case group. The following exclusion criteria were applied: (1) use of oral contraceptives or exogenous hormonal medications within the past three months; (2) chromosomal abnormalities; (3) had a history of ovarian surgery, endometriosis, endocrine disorders including polycystic ovary syndrome, thyroid disorders, diabetes, Cushing’s syndrome, hyperprolactinemia or a major chronic disease. The control group included women with normal ovarian reserve who presented during the same period for infertility evaluation due to tubal factors or male partner-related causes. The exclusion criteria were the same as those applied to the case group. A total of 4,231 DOR cases and 19,071 controls were included. Controls were exactly matched to cases precisely in a 1:1 ratio based on age (n=3751) (**Figure 1**).

### Exposure and laboratory assessment

Basic demographic and clinical data were extracted from the electronic medical records, including age, ethnicity, occupation, marital status, educational level, smoking and alcohol consumption, height, weight, blood type, menstrual history, reproductive history, previous surgical history, genetic and family history, and microbial infections. Baseline sex hormone levels were measured using a chemiluminescence immunoassay (CLIA) on days 2 to 4 of the menstrual cycle, including AMH, follicle-stimulating hormone (FSH), estradiol (E2), progesterone (P), prolactin (PRL), luteinizing hormone (LH), and total testosterone (T). AFC was measured underwent transvaginal ultrasonography to evaluate the total number of antral follicles in both ovaries. Serological testing for TORCH infections was performed using chemiluminescence immunoassay (CLIA). The definition of a positive “infection” status was specific for each pathogen, based on the presence of immunoglobulin M (IgM) antibodies, which typically indicate recent or active infection. Given the high prevalence of rubella vaccination, infection was strictly defined as a positive result for rubella-specific IgM. The presence of rubella IgG alone was interpreted as evidence of immunity and not considered an active infection for the purpose of this study.

Cervical samples for HPV detection were obtained outside the menstrual period. Participants were instructed to avoid vaginal douching and sexual intercourse for at least 48 hours prior to sampling. Exfoliated cervical epithelial cells were collected by inserting a cervical brush into the endocervical canal and rotating it clockwise five times. Samples were immediately placed into tubes containing HPV preservation medium and tested using a validated HPV DNA genotyping assay capable of detecting 26 HPV genotypes.

BMI was calculated as weight (kg) divided by height (m)^2, which was further classified into four groups: underweight (BMI <18.5 kg/m²), normal weight (18.5 ≤ BMI ≤ 23.9 kg/m²), overweight (24 ≤ BMI ≤ 27.9 kg/m²), and obese (BMI >28 kg/m²) (17).

### Statistical analyses

For descriptive statistics, categorical variables were expressed as n (%), and between group comparisons were performed using the McNemar’s tests. Since none of the continuous variables followed a normal distribution by Kolmogorov-Smirnov test, they were reported as median values with inter-quartile range (M [IQR]), and comparisons between groups were performed using the Wilcoxon signed-rank test for paired samples. Univariate conditional logistic regression analyses were performed to screen for candidate variables significantly associated with DOR (P<0.05). Variables with statistical significance in the univariate analyses were subsequently included in a multivariate conditional logistic regression model to adjust for potential confounders and to identify independent risk factors. Subgroup analyses were conducted across different age groups (20–35 and 36–47 years) and BMI strata, two well-known predictors of ovarian reserve function (3, 18, 19). For the purpose of effect modification analyses, BMI was further dichotomized using a cut-off of 24 kg/m² (<24 and ≥24 kg/m²) to ensure adequate sample size within strata and to avoid sparse data and model instability (20). Multivariate conditional logistic regression models were constructed within each subgroup to evaluate whether the associations remained significant. Interaction terms were generated using cross-product terms, and P-values for interaction were calculated to examine potential effect modification. All statistical analyses were performed using R software version 4.4.2, with a two-tailed *P*-value <0.05 considered as a statistically significant threshold.

## Results

### Baseline characteristics

The baseline characteristics of 7,502 participants are shown in **Table 1**. The median age was 36 years (IQR:32-39) years, and the participants aged 20–35 years accounted for half of the total number. In total, 85.8% of the participants were of Han ethnicity, and the vast majority were married women (97.3%). Manual laborers accounted for 44.3% of the cohort, while 10.3% were engaged in mental labor. The median BMI was 22.15 (IQR: 20.31–24.44), with most participants having a normal weight (63.7%) and 4.5% classified as obese. The majority of participants were non-smokers (94.8%) and non-drinkers (99.8%). Most women reported normal menstrual volume (81.6%) and no significant dysmenorrhea (81.1%). Over half of the women (58%) had fewer than two pregnancies, and 94.5% had fewer than two deliveries. Additionally, 14.9% had undergone two or more induced abortions.

**Table 1:** Baseline characteristics of 7502 women enrolled in the study ^a,c^.

Characteristics	Total (n=7502)	Case (n=3751)	Control (n=3751)	*P* value
Age(y), M(IQR)	36(32~39)			
20-35	3704(49.4)	1852(49.4)	1852(49.4)	
36-47	3798(50.6)	1899(50.6)	1899(50.6)	
Ethnicity, n (%)				<0.001
Han	6437(85.8)	3160(84.2)	3277(87.4)	
Non-Han	1065(14.2)	591(15.8)	474(12.6)	
Marital status, n (%)				0.278
Married	6713(97.3)	3377(97)	3336(97.7)	
Single	155(2.2)	87(2.5)	68(2)	
Divorced	28(0.5)	16(0.5)	12(0.4)	
Education, n (%)				0.687
High school or below	4814(64.2)	2399(64)	2415(64.4)	
Undergraduate	2480(33)	1242(33.1)	1238(33)	
Postgraduate	208(2.8)	110(2.9)	98(2.6)	
Occupation ^b^, n (%)				0.017
Other	3408(45.4)	1712(45.7)	1702(45.4)	
Manual labor	3320(44.3)	1618(43.1)	352(9.4)	
Non-manual labor	772(10.3)	420(11.2)	1696(45.2)	
**BMI (kg/m^2^), M(IQR)**	22.15 (20.31~24.44)	22.22 (20.34~24.44)	22.06 (20.31~24.41)	**0.023**
BMI (kg/m^2^), n (%)				0.016
18.5-23.9	4781(63.7)	2384(63.6)	2397(63.9)	
<18.5	518(6.9)	243(6.5)	275(7.3)	
24-27.9	1866(24.9)	929(24.8)	937(25)	
≥28	337(4.5)	195(5.2)	142(3.8)	
Smoking, n (%)				0.614
No	6690(94.8)	3197(94.7)	3493(95)	
Yes	364(5.2)	179(5.3)	185(5)	
Alcohol drinking, n (%)				0.302
No	7483(99.8)	3738(99.7)	3745(99.9)	
Yes	15(0.2)	10(0.3)	5(0.1)	
ABO blood type, n (%)				0.301
A	2436(32.6)	1184(31.7)	1252(33.5)	
B	1855(24.9)	955(25.6)	900(24.1)	
O	2559(34.3)	1287(34.5)	1272(34.1)	
AB	613(8.2)	304(8.2)	309(8.3)	
Menstrual flow, n (%)				0.004
Moderate	6123(81.6)	3008(80.2)	3115(83)	
Light	1240(16.5)	673(18)	567(15.1)	
Heavy	137(1.8)	68(1.8)	69(1.8)	
Dysmenorrhea, n (%)				0.906
No	6083(81.1)	3038(81)	3045(81.2)	
Yes	1417(18.9)	711(19)	706(18.8)	
No. pregnancies, n (%)				<0.001
<2	4351(58)	2256(60.1)	2095(55.9)	
≥2	3151(42)	1495(39.9)	1656(44.1)	
No. productions, n (%)				0.798
<2	7091(94.5)	3549(94.6)	3542(94.5)	
≥2	410(5.5)	202(5.4)	208(5.5)	
No. induced abortions, n (%)				0.070
<2	6381(85.1)	3220(85.8)	3161(84.3)	
≥2	1120(14.9)	531(14.2)	589(15.7)	
HPV infection, n (%)				0.527
No	6949(93.9)	3474(93.8)	3475(94.1)	
Yes	448(6.1)	230(6.2)	218(5.9)	
Toxoplasma gondii infection, n (%)				<0.001
No	6637(88.6)	3169(84.6)	3468(92.5)	
Yes	856(11.4)	576(15.4)	280(7.5)	
Cytomegalovirus infection, n (%)				<0.001
No	6510(86.9)	3116(83.2)	3394(90.5)	
Yes	984(13.1)	629(16.8)	355(9.5)	
Rubella virus infection, n (%)				<0.001
No	6496(86.7)	3106(83)	3390(90.4)	
Yes	997(13.3)	638(17)	359(9.6)	
HSV infection, n (%)				0.404
No	7302(97.6)	3653(97.7)	3649(97.4)	
Yes	182(2.4)	85(2.3)	97(2.6)	

M(IQR), median (inter-quartile range); BMI, body mass index; HPV, Human Papillomavirus; HSV, Herpes Simplex Virus.

a. Data presented as M(IQR) or number (%) as appropriate. Total numbers may not sum to 7,502 due to missing data in some variables and percentages may not sum to 100% due to rounding.

b. Manual labor includes agricultural and other physical job; non-manual labor includes office or professional work; and other includes students, retirees, and the unemployed.

c. Category counts do not always sum to the total number of participants because of missing data in certain variables. Percentages are calculated based on non-missing values.

Bold values indicate statistical significance at p < 0.05.

Regarding microbial infections, a minority of participants tested positive for human papillomavirus (HPV), Toxoplasma gondii (T. gondii), cytomegalovirus (CMV), rubella virus (RV), and herpes simplex virus (HSV), with infection rates of 6.1%, 11.4%, 13.1%, 13.3%, and 2.4%, respectively.

When comparing the distribution of variables between the case and control groups, no significant differences were observed in marital status, education level, smoking and alcohol history, ABO blood type, dysmenorrhea, number of deliveries and induced abortions, or HPV infection. Compared to controls, cases had a higher BMI (22.22 vs. 22.06) kg/m^2^, a higher proportion of non-Han ethnicity (15.8% vs. 12.6%, *P* < 0.001), and more participants engaged in manual labor (43.1% vs. 9.4%, *P* < 0.05). Additionally, cases were more likely to report light menstrual flow volume (18% vs. 15.1%, *P* < 0.05) and fewer than two pregnancies (60.1% vs. 55.9%, *P* < 0.001). Infections with T. gondii, CMV, and RV were significantly more common among cases group (*P* < 0.001).

### Reproductive hormones and AFC comparison

**Table 2** shows the reproductive hormones and AFC of the two groups. SignIficant differences were observed in the AMH, FSH, E2, PRL, LH, T and AFC(*P* < 0.01). Specifically, cases had higher FSH (9.14 IU/L vs. 7.22 IU/L, *P* < 0.001) and slightly higher E2 and LH levels (33 pg/mL vs. 32 pg/mL, P<0.001;3.77 IU/L vs.3.74 IU/L, P<0.01). Conversely, they had lower PRL (236.34 IU/L vs. 251.17 IU/L, *P* < 0.001) and T (26.48 ng/dL vs. 32.92 ng/dL, *P* < 0.001) levels. Additionally, AFC and AMH levels were significantly lower in the DOR group (AFC: 5 vs. 15, *P* < 0.001; AMH: 0.68 ng/mL vs. 2.86 ng/mL, *P* < 0.001). P levels did not differ between groups (p=0.662).

**Table 2:** Reproductive hormones and antral follicle count in case and control group.

Reproductive hormones	Total(n=7502)	Case(n=3751)	Control(n=3751)	*P* value
AMH (ng/ml), M(IQR)	1.38 (0.68~2.88)	0.68(0.38~0.94)	2.86(1.94~4.35)	<0.001
FSH (IU/L), M(IQR)	7.88 (6.4~10.12)	9.14(7~12.44)	7.22(6.08~8.5)	<0.001
E2 (pg/ml), M(IQR)	32.54 (24.49~44)	33(23~46)	32(25.9~41.89)	<0.001
P (ng/ml), M(IQR)	0.46 (0.3~0.7)	0.46(0.29~0.71)	0.46(0.31~0.7)	0.662
PRL (IU/L), M(IQR)	243.62 (167.97~341.63)	236.34(158.87~335.84)	251.17(176.26~346.97)	<0.001
LH(IU/L), M(IQR)	3.74 (2.72~5.11)	3.77(2.68~5.29)	3.74(2.77~4.93)	0.002
T (ng/dL), M(IQR)	29.68 (14.46~43.61)	26.48(10.51~40.63)	32.92(18.77~46.09)	<0.001
AFC (n), M(IQR)	9 (5~15)	5(3~8)	15(10~20)	<0.001

M(IQR), median (inter-quartile range); AMH, anti-müllerian hormone; FSH, follicle-stimulating hormone; E2, estradiol (Estradiol-17β); P, progesterone; PRL, prolactin; LH, luteinizing hormone; T, testosterone; AFC, antral follicle count.

### Univariate and multivariable logistic regression analysis

The results of univariate and multivariate logistic regression analyses of potential factors associated with DOR are presented in **Table 3**. In the univariate model, non-Han ethnicity (OR, 1.293; 95%CI, 1.134-1.473), manual labor (OR, 1.258; 95%CI, 1.074-1.474), obesity (OR, 1.394; 95%CI, 1.112-1.747), and light menstrual flow (OR, 1.235; 95%CI, 1.090-1.398) and having two or more pregnancies (OR, 0.826; 95%CI, 0.750-0.909) were significantly associated with DOR. Regarding microbial infections, T. gondii (OR, 2.333; 95%CI, 1.994-2.731), CMV (OR, 1.975; 95%CI, 1.711-2.280), and RV infections (OR, 1.986; 95%CI, 1.721-2.291) were also associated with DOR.

**Table 3:** Conditional logistic regression results for the association between the risk factors with DOR.

Variables	Univariable logistic regression			Multivariable logistic regression ^a^		
	OR	95% CI	*P* value	OR	95% CI	*P* value
Ethnicity			<0.001			<0.001
Han	ref			ref		
Non-Han	1.293	1.134-1.473		1.278	1.115-1.466	
Occupation ^b^						
Other	ref			ref		
Manual labor	1.258	1.074-1.474	0.004	1.181	1.002-1.392	**0.047**
Non-manual labor	1.061	0.964-1.168	0.227	1.053	0.953-1.163	0.314
BMI						
18.5-23.9	ref			ref		
<18.5	0.884	0.737-1.060	0.183	0.884	0.733-1.065	0.193
24-27.9	0.995	0.895-1.107	0.931	0.949	0.850-1.059	0.349
≥28	1.394	1.112-1.747	0.004	1.316	1.044-1.660	**0.020**
Menstrual flow						
Moderate	ref			ref		
Light	1.235	1.090-1.398	<0.001	1.262	1.111-1.435	**<0.001**
Heavy	1.020	0.727-1.430	0.910	1.041	0.737-1.471	0.821
No. pregnancies			<0.001			<0.001
<2	ref			ref		
≥2	0.826	0.750-0.909		0.830	0.751-0.916	
Toxoplasma gondii infection			<0.001			<0.001
No	ref			ref		
Yes	2.333	1.994-2.731		2.292	1.683-3.122	
Cytomegalovirus infection			<0.001			0.343
No	ref			ref		
Yes	1.975	1.711-2.280		0.695	0.327-1.475	
Rubella virus infection			<0.001			0.301
No	ref			ref		
Yes	1.986	1.721-2.291	0.000	1.474	0.706-3.080	

CI, confidence interval; OR, odds ratio; BMI, body mass index.

a. The selection of covariates for multivariable conditional logistic regression was based on a univariable screening threshold of p<0.05.

b. Manual labor includes agricultural and other physical job; non-manual labor includes office or professional work; and other includes students, retirees, and the unemployed.

Bold values indicate statistical significance at p < 0.05.

After adjusting for potential confounders, the multivariate logistic regression model revealed that non-Han ethnicity (OR, 1.278; 95%CI, 1.115-1.466), manual labor (OR, 1.181; 95%CI, 1.002-1.392), obesity (OR, 1.316; 95%CI, 1.044-1.660), light menstrual flow (OR, 1.262; 95%CI, 1.111-1.435), and T. gondii infection (OR, 2.292; 95%CI, 1.683-3.122) were independently associated with DOR. Conversely, having two or more pregnancies (OR, 0.830; 95%CI, 0.751-0.916) was inversely associated with the DOR.

### Subgroup analysis of results stratified by age and BMI

The results from subgroup analyses stratified by age and BMI are shown in **Tables 4**, **5**. In the subgroup analysis stratified by age, multivariate logistic regression indicated that non-Han ethnicity (OR, 1.449; 95%CI, 1.197-1.754), obesity (OR, 1.480; 95%CI, 1.064-2.060), and manual labor occupation (OR, 1.552; 95%CI, 1.196-2.015) were significantly associated with DOR among women aged 20–35 years, whereas these associations were not observed in the 36–47 years group. However, light menstrual flow (20–35 years: OR, 1.290; 95%CI, 1.071-1.553;36–47 years: OR, 1.190; 95%CI, 1.008-1.408) remained significantly associated with DOR in both age groups. Subgroup analysis showed no significant interaction between age and these factors on DOR (*P* for interaction>0.05). In contrast, having two or more pregnancies (OR, 0.712; 95%CI, 0.615-0.824), as well as infection with T. gondii (OR, 23.750; 95%CI, 13.330-42.316), CMV (OR, 8.189; 95%CI, 5.821-11.521), and RV (OR, 8.132; 95%CI, 5.806-11.390) were significantly associated with DOR in the 20–35 years group but not in the 36–47 years group. The association between these factors and DOR differed by age group (*P* for interaction < 0.05). (**Table 4**).

**Table 4:** The association between the risk factors with DOR stratified by age based on conditional logistic regression models ^a^.

Variables		N		20–35 years		N		36–47 years		
		Case	Control	OR	95%CI	Case	Control	OR	95%CI	*P* for interaction
Ethnicity	Han	1565	1645	ref		1595	1632	ref		
	Non-Han	287	207	**1.449**	**1.197-1.754**	304	267	1.167	0.975-1.396	0.180
BMI	18.5-23.9	1209	1222	ref		1175	1175	ref		
	<18.5	159	177	0.903	0.720-1.134	84	98	0.851	0.627-1.155	0.829
	24-27.9	392	390	1.020	0.869-1.198	537	547	0.975	0.845-1.125	0.355
	≥28	92	63	**1.480**	**1.064-2.060**	103	79	1.322	0.970-1.801	0.768
Occupation	Other	814	872	ref		804	830	ref		
	Manual labor	166	116	**1.552**	**1.196-2.015**	254	236	1.111	0.908-1.359	0.094
	Non-manual labor	872	863	1.076	0.941-1.232	840	833	1.043	0.909-1.197	0.998
Menstrual flow	Moderate	1510	1570	ref		1498	1545	ref		
	Light	302	245	**1.290**	**1.071-1.553**	371	322	**1.191**	**1.008-1.408**	0.862
	Heavy	38	37	1.062	0.675-1.672	30	32	0.969	0.583-1.610	0.819
No. pregnancies	<2	1382	1257	ref		874	838	ref		
	≥2	470	595	**0.712**	**0.615-0.824**	1025	1061	0.927	0.815-1.053	**0.006**
Toxoplasma gondii infection	No	1559	1838	ref		1610	1630	ref		
	Yes	287	14	**23.750^b^**	**13.330-42.316**	289	266	1.110	0.921-1.337	**<0.001**
Cytomegalovirus infection	No	1539	1811	ref		1577	1583	ref		
	Yes	307	41	**8.189**	**5.821-11.521**	322	314	1.033	0.866-1.232	**<0.001**
Rubella virus infection	No	1533	1810	ref		1573	1580	ref		
	Yes	313	42	**8.132**	**5.806-11.390**	325	317	1.033	0.866-1.231	**<0.001**

BMI, body mass index.

a. All variables meeting the prescreening threshold of p<0.05 in univariable analysis were retained for multivariable conditional logistic regression, with subsequent stratification by age.

b. The odds ratios for the 20–35 years age subgroup are based on a limited number of exposed subjects and should be interpreted as exploratory findings due to potential instability from sparse data.

Bold values indicate statistical significance at p < 0.05

**Table 5:** The association between the risk factors with DOR stratified by BMI based on conditional logistic regression models ^a^.

Variable		N		BMI <24 kg/m^2^		N		BMI ≥24 kg/m^2^		P for interaction
		Case	Control	OR	95%CI	Case	Control	OR	95%CI	
Ethnicity	Han	2281	2379	ref		879	898	ref		
	Non-Han	346	293	**1.264**	**1.040-1.535**	245	181	**1.541**	**1.019-2.330**	0.317
Occupation	Other	1122	1179	ref		496	523	ref		
	Manual labor	246	198	1.231	0.962-1.575	174	154	1.391	0.848-2.284	0.448
	Non-manual labor	1259	1295	1.054	0.921-1.206	453	401	1.113	0.784-1.580	0.213
Menstrual flow volume	Moderate	2110	2214	ref		898	901	ref		
	Light	476	419	**1.229**	**1.033-1.461**	197	148	1.328	0.853-2.066	0.315
	Heavy	39	39	1.048	0.605-1.816	29	30	0.829	0.284-2.421	0.934
No. pregnancies	<2	1600	1517	ref		656	578	ref		
	≥2	1027	1155	**0.807**	**0.703-0.926**	468	501	0.831	0.611-1.132	0.963
Toxoplasma gondii infection	No	2234	2495	ref		935	973	ref		
	Yes	389	174	**2.567**	**2.030-3.246**	187	106	**1.958**	**1.198-3.202**	0.088
Cytomegalovirus infection	No	2196	2446	ref		920	948	ref		
	Yes	427	224	**2.103**	**1.701-2.600**	202	131	1.361	0.885-2.093	0.070
Rubella virus infection	No	2189	2442	ref		917	948	ref		
	Yes	434	228	**2.159**	**1.748-2.666**	204	131	1.389	0.905-2.132	0.070

a. All variables meeting the prescreening threshold of p<0.05 in univariable analysis were retained for multivariable conditional logistic regression, with subsequent stratification by BMI.

Bold values indicate statistical significance at p < 0.05

In the BMI-stratified subgroup analysis, non-Han ethnicity (BMI <24 kg/m^2^: OR, 1.264; 95%CI, 1.040-1.535; BMI ≥24 kg/m^2^, OR, 1.541; 95%CI, 1.019-2.330) and T. gondii infection (BMI <24 kg/m^2^: OR, 2.567; 95%CI, 2.030-3.246; BMI ≥24 kg/m^2^, OR, 1.958; 95%CI, 1.198-3.202) were significantly associated with DOR in both BMI subgroups. Light menstrual flow (OR, 1.229; 95%CI, 1.033-1.461), having two or more pregnancies (OR, 0.807; 95%CI, 0.703-0.926), and CMV (OR, 2.103; 95%CI, 1.701-2.600) and RV infections (OR, 2.159; 95%CI, 1.748-2.666) were significantly associated with DOR in women with BMI <24 kg/m², but not in those with BMI ≥24 kg/m². However, no statistically significant interaction was observed (*P* for interaction>0.05). (**Table 5**).

## Discussion

To our knowledge, this is the first large-scale, age-matched case-control study investigating DOR in Chinese women. We identified significant associations between DOR and factors including ethnicity, occupation, obesity, light menstrual flow, two or more pregnancies, and T. gondii infection. Notably, the association between pregnancies and infection with T. gondii, CMV, and RV infections with DOR varied across age groups, with evidence of significant effect modification by age. These findings suggest new links between TORCH infections and DOR, and highlight the need for age-specific prevention strategies.

FSH, AFC, and AMH are commonly used in ovarian reserve examinations. AMH is primarily secreted by primary, preantral, and early antral follicles. With advancing age, the number of ovarian follicles declines, leading to decreased concentrations of AMH (5, 21). FSH secretion is regulated by the hypothalamic–pituitary–ovarian (HPO) axis. Under normal ovarian function, sufficient levels of E2 and inhibin B are produced to exert negative feedback on FSH secretion, maintaining it within the normal range. Elevated FSH levels indicate DOR, suggesting impaired ovarian feedback and insufficient hormonal suppression of FSH (5). Importantly, measurement of both FSH and E2 on cycle day 3 may help decrease the incidence of false-negative testing (6). AFC is the sum of the number of antral follicles in both ovaries during the early-follicular phase. with a higher count generally indicating preserved ovarian function. In our study, significant differences were determined in all measures of ovarian reserve between two groups, and DOR group exhibited significantly lower AMH, AFC, and T levels, and higher FSH, LH and E2 levels. In women with DOR, impaired negative feedback regulation may lead to elevated LH secretion. The reduction in follicle number and the decline in the functional capacity of the theca cells may result in decreased testosterone levels. There were no significant differences in progesterone levels measured during the early follicular phase. This finding is biologically plausible, as progesterone secretion during this phase is minimal and primarily reflects the absence of luteal activity rather than ovarian reserve status. Unlike anti-Müllerian hormone (AMH) and follicle-stimulating hormone (FSH), which directly reflect follicular quantity and responsiveness, basal progesterone is not considered a sensitive marker of diminished ovarian reserve (6, 22). In addition, consistent with previous studies (23), participants with DOR exhibited lower serum PRL levels, which may reflect an overall alteration in the function of the HPO axis. This suggests that lower prolactin levels are likely a feedback result of diminished ovarian reserve or a reduced follicular pool, rather than a causative factor. PRL secretion is influenced by various factors such as emotions and stress (24). The present study excluded patients with hyperprolactinemia, and the observed differences fell within the low end of the normal reference range. This indicates that when evaluating ovarian reserve, attention should not only be paid to elevated prolactin levels; lower levels may also be associated with ovarian functional status.

Data from Carlos I et al. indicate that ethnicity may influence ovarian reserve, with Latina and Chinese women exhibiting lower AMH levels compared to White women, suggesting DOR and a higher risk of earlier menopause (25, 26). Our findings extend this observation, demonstrating that ethnic disparities may exist even within the same racial group (East Asians), as evidenced by differences between Han and minority populations in China. Further longitudinal studies are needed to clarify whether these differences are driven by genetic, nutritional, environmental, or lifestyle factors, and to elucidate the underlying mechanisms for better prediction of reproductive potential and long-term health outcomes. Ethnicity also modulates the association between obesity and ovarian reserve. Compared to Asian and Hispanic women, White women exhibit higher baseline AMH levels, with elevated BMI further reducing AMH levels in White and African-American women but not in Asian or Hispanic populations (13, 27, 28). Notably, variations in obesity classification criteria may contribute to these disparities. While BMI is widely used to assess health risks, its optimal thresholds differ across ethnic groups. For a given BMI, non-Hispanic Asians have more body fat than non-Hispanic Whites (29). In our study, adopting the Chinese BMI criteria (obese defined as BMI ≥28 kg/m²), we identified obesity as a significant risk factor for DOR, consistent with findings reported by Li YL et al. and Moslehi N et al. (30, 31). Regular physical activity helps to mitigate the tendency for weight gain and adverse changes in body composition and fat distribution that accompany aging and the menopausal transition (32). However, women engaging in high-intensity physical activity or demanding occupational labor exhibit reduced fertility, manifested by fewer retrieved mature oocytes following controlled ovarian hyperstimulation, as demonstrated in our study (33).

Previous studies have identified light menstrual flow as a risk factor for infertility, whereas number of pregnancies serves as a protective factor (2, 34). Our findings demonstrate similar associations between menstrual flow, pregnancy, and DOR. In addition, a study investigating the effects of reproductive and lifestyle factors on age-specific AMH levels reported that pregnant women exhibited significantly lower AMH concentrations, while higher parity was associated with higher AMH (35). Currently, no conclusive evidence suggests that pregnancy accelerates or slows the long-term decline of ovarian reserve. Whether multiple pregnancies exert a protective effect on ovarian reserve by reducing the number of ovulatory cycles and subsequent follicle depletion, or whether the observed association reflects a reverse causality—where DOR leads to reduced fertility and thus fewer pregnancies—remains uncertain and warrants cautious interpretation. Light menstrual flow may also represent a clinical manifestation of advanced ovarian reserve decline. DOR can lead to a shortened follicular phase, reduced estrogen peak levels, inadequate endometrial proliferation, and consequently, decreased menstrual volume. However, due to study design limitations, causal inferences remain constrained, warranting further prospective research to elucidate the underlying mechanisms. Nevertheless, persistent reductions in menstrual flow in women under 35 years should raise clinical suspicion for premature ovarian insufficiency or early ovarian aging, necessitating prompt ovarian reserve assessment.

TORCH infections are known to cause adverse pregnancy outcomes such as miscarriage, preterm birth, and congenital anomalies (36, 37). In our study, the seroprevalence of T. gondii (11.4%) was higher than that reported in the general population of women of reproductive age. Given that the participants were from Southwest China—a region with the highest ethnic diversity in the country—this finding may reflect a distinct regional epidemiological context, including local dietary practices, environmental exposures, and pathogen circulation. In addition, this elevated prevalence may partly relate to the characteristics of the study population, which consisted of women seeking fertility evaluation and therefore may differ from the general population. Our findings reveal a significant association between T. gondii infection and DOR. Notably, stratified analyses by age exhibited TORCH infections (including T. gondii, CMV and RV) significantly related to DOR among aged <35 years subgroup, whereas no significant correlation was observed in older women. This age-dependent interaction was significant, possibly because the physiological decline in ovarian reserve with age overshadows the effects of infection. Additionally, younger women may exhibit a more pronounced or sustained immune response to infections, leading to greater ovarian tissue damage. However, it remains unclear whether the observed associations are attributable to primary infections or to reactivation. Notably, the very large odds ratios observed for TORCH infections, particularly T. gondii (OR = 23.750), in women aged 20–35 years were accompanied by wide confidence intervals. This likely reflects sparse-data bias arising from the small number of exposed controls in this subgroup, resulting in limited precision and instability of the effect estimates. Accordingly, these findings should be interpreted as signals of potential association rather than as precise estimates of effect magnitude. In BMI-stratified analysis, the association between TORCH infection and DOR was evident among BMI ≤24 kg/m^2^ subgroup; however, no significant interaction was detected, indicating that BMI may not substantially modify this relationship. TORCH infections may contribute to DOR through immune activation, inflammatory responses, and direct ovarian tissue damage (38, 39). Whilst HSV is renowned for its tropism towards genital tract epithelium and can cause pelvic inflammatory disease, its primary pathology is typically focal and recurrent (40). In contrast, pathogens such as Toxoplasma gondii or rubella virus cause systemic infections, potentially exerting broader immunopathological or direct cytotoxic effects on ovarian tissue. Alternatively, the lack of association may stem from timing issues in our assessment; common HSV reactivation may not be adequately captured by a single IgM test, or its impact on ovarian reserve may be subtle. Overall, TORCH screening may be considered for younger women with unexplained DOR, particularly in regions with a high prevalence of these infections. However, it must be emphasized that our results do not provide evidence that antimicrobial or antiviral treatment targeting these infections can reverse or improve ovarian reserve. Whether pathogen-specific interventions can modify ovarian function remains a fundamental question that warrants mechanistic investigations and prospective epidemiological studies.

The key strengths of this study include the adequate sample size and age-matched case-control design, which enhanced study efficiency. Nonetheless, several limitations should be acknowledged. First, residual confounding may persist due to the lack of data on environmental exposures, dietary habits, and nutritional supplementation. Second, the assessment of certain clinical variables, such as “menstrual flow”, was based on patient self-report documented in electronic medical records without a standardized quantitative definition. This reliance on subjective recall introduces the potential for misclassification and recall bias, which may have attenuated the observed associations for this factor. Third, TORCH infections in this study were assessed based on serological IgM positivity. Although IgM testing increases specificity for recent or active infection, it does not allow precise determination of the timing of exposure and may also reflect persistent antibodies or pathogen reactivation. In the absence of longitudinal serological follow-up or documented infection history, the biological interpretation of infection-related ovarian impairment should therefore be considered speculative. Additionally, since all participants were recruited from a maternity hospital, and the control group included women with underlying gynecological conditions such as tubal obstruction and endometrial polyps, caution should be exercised when generalizing the findings to the general population. Due to the observational nature of the study, additional limitations such as multiple testing, measurement bias, and temporal ambiguity cannot be ruled out. Future large-scale prospective cohort or experimental studies are needed to further investigate the determinants and underlying mechanisms of ovarian reserve and to validate our findings.

## Conclusions

In summary, this large age-matched case-control study identified multiple clinical and demographic factors associated with DOR, including ethnicity, obesity, menstrual flow, pregnancy history, and TORCH infections, with evidence of age-related effect modification for pregnancy history and infection. While the study provides novel insights into risk stratification, limitations such as potential residual confounding and diagnostic variability highlight the need for standardized DOR criteria and prospective investigations. Future research should prioritize elucidating causal mechanisms—particularly the role of infections and immune responses—and validate these associations in diverse populations to inform clinical screening and preventive strategies for at-risk women.

## Data Availability

The original contributions presented in the study are included in the article/supplementary material. Further inquiries can be directed to the corresponding author.
